# Supplementary material for: Dual-branch differential channel hypergraph convolutional network for human skeleton based action recognition
Source: PLoS One. 2025 Oct 27;20(10):e0332066. doi: 10.1371/journal.pone.0332066 (PMC12558502; doi:10.1371/journal.pone.0332066)
Supplement: S1 File — It briefly contains the code of the key modules of the neural network in this experiment. (PDF) [file pone.0332066.s001.pdf]

```
import math
import pdb
from graph import hypergraph_utils as hgut
import numpy as np
import torch
import torch.nn as nn
from torch.autograd import Variable
import torch.nn.functional as F

#from torchstat import stat
#from thop import profile

iii=0
from sklearn.metrics import pairwise_distances
import scipy.sparse as sparse


class AFF(nn.Module):
    '''

    '''

    def __init__(self, channels=64, r=4):
        super(AFF, self).__init__()
        inter_channels = int(channels // r)

        self.local_att = nn.Sequential(
            nn.Conv2d(channels, inter_channels, kernel_size=1, stride=1, padding=0),
            nn.BatchNorm2d(inter_channels),
            nn.ReLU(inplace=True),
            nn.Conv2d(inter_channels, channels, kernel_size=1, stride=1, padding=0),
            nn.BatchNorm2d(channels),
        )

        self.global_att = nn.Sequential(
            nn.AdaptiveAvgPool2d(1),
            nn.Conv2d(channels, inter_channels, kernel_size=1, stride=1, padding=0),
            nn.BatchNorm2d(inter_channels),
            nn.ReLU(inplace=True),
            nn.Conv2d(inter_channels, channels, kernel_size=1, stride=1, padding=0),
            nn.BatchNorm2d(channels),
        )

        self.sigmoid = nn.Sigmoid()
```

```
    def forward(self, x, residual):
        xa = x + residual
        xl = self.local_att(xa)
        xg = self.global_att(xa)
        xlg = xl + xg
        wei = self.sigmoid(xlg)

        xo = 2 * x * wei + 2 * residual * (1 - wei)
        return xo


#import hypergraph_utils as hgut
def import_class(name):
    components = name.split('.')
    mod = __import__(components[0])
    for comp in components[1:]:
        mod = getattr(mod, comp)
    return mod


def conv_branch_init(conv, branches):
    weight = conv.weight
    n = weight.size(0)
    k1 = weight.size(1)
    k2 = weight.size(2)
    nn.init.normal_(weight, 0, math.sqrt(2. / (n * k1 * k2 * branches)))
    nn.init.constant_(conv.bias, 0)


def conv_init(conv):
    if conv.weight is not None:
        nn.init.kaiming_normal_(conv.weight, mode='fan_out')
    if conv.bias is not None:
        nn.init.constant_(conv.bias, 0)


def bn_init(bn, scale):
    nn.init.constant_(bn.weight, scale)
    nn.init.constant_(bn.bias, 0)


def weights_init(m):
    classname = m.__class__.__name__
```

```
        if classname.find('Conv') != -1:
            if hasattr(m, 'weight'):
                nn.init.kaiming_normal_(m.weight, mode='fan_out')
            if hasattr(m, 'bias') and m.bias is not None and isinstance(m.bias, torch.Tensor):
                nn.init.constant_(m.bias, 0)
        elif classname.find('BatchNorm') != -1:
            if hasattr(m, 'weight') and m.weight is not None:
                m.weight.data.normal_(1.0, 0.02)
            if hasattr(m, 'bias') and m.bias is not None:
                m.bias.data.fill_(0)


class TemporalConv(nn.Module):
    def __init__(self, in_channels, out_channels, kernel_size, stride=1, dilation=1):
        super(TemporalConv, self).__init__()
        pad = (kernel_size + (kernel_size-1) * (dilation-1) - 1) // 2
        self.conv = nn.Conv2d(
            in_channels,
            out_channels,
            kernel_size=(kernel_size, 1),
            padding=(pad, 0),
            stride=(stride, 1),
            dilation=(dilation, 1))

        self.bn = nn.BatchNorm2d(out_channels)

    def forward(self, x):
        x = self.conv(x)
        x = self.bn(x)
        return x


class MultiScale_TemporalConv(nn.Module):
    def __init__(self,
                 in_channels,
                 out_channels,
                 kernel_size=3,
                 stride=1,
                 dilations=[1,2,3,4],
                 residual=True,
                 residual_kernel_size=1):

        super().__init__()
        assert out_channels % (len(dilations) + 2) == 0, '# out channels should be multiples of #
```

branches'

```
        # Multiple branches of temporal convolution
        self.num_branches = len(dilations) + 2
        branch_channels = out_channels // self.num_branches
        if type(kernel_size) == list:
            assert len(kernel_size) == len(dilations)
        else:
            kernel_size = [kernel_size]*len(dilations)
        # Temporal Convolution branches
        self.branches = nn.ModuleList([
            nn.Sequential(
                nn.Conv2d(
                    in_channels,
                    branch_channels,
                    kernel_size=1,
                    padding=0),
                nn.BatchNorm2d(branch_channels),
                nn.ReLU(inplace=True),
                TemporalConv(
                    branch_channels,
                    branch_channels,
                    kernel_size=ks,
                    stride=stride,
                    dilation=dilation),
            )
            for ks, dilation in zip(kernel_size, dilations)
        ])

        # Additional Max & 1x1 branch
        self.branches.append(nn.Sequential(
            nn.Conv2d(in_channels, branch_channels, kernel_size=1, padding=0),
            nn.BatchNorm2d(branch_channels),
            nn.ReLU(inplace=True),
            nn.MaxPool2d(kernel_size=(3,1), stride=(stride,1), padding=(1,0)),
            nn.BatchNorm2d(branch_channels)    #
        ))

        self.branches.append(nn.Sequential(
            nn.Conv2d(in_channels,    branch_channels,    kernel_size=1,    padding=0,
stride=(stride,1)),
            nn.BatchNorm2d(branch_channels)
        ))
```

```
        # Residual connection
        if not residual:
            self.residual = lambda x: 0
        elif (in_channels == out_channels) and (stride == 1):
            self.residual = lambda x: x
        else:
            self.residual = TemporalConv(in_channels, out_channels,
kernel_size=residual_kernel_size, stride=stride)

        # initialize
        self.apply(weights_init)
        self.af=AFF(out_channels)
    def forward(self, x):
        # Input dim: (N,C,T,V)
        res = self.residual(x)
        branch_outs = []
        for tempconv in self.branches:
            out = tempconv(x)
            branch_outs.append(out)

        out = torch.cat(branch_outs, dim=1)
        aff=self.af
        out=aff(out,res)
      #   out += res
        return out

class CTRGC(nn.Module):
    def __init__(self, in_channels, out_channels, rel_reduction=8, mid_reduction=1):
        super(CTRGC, self).__init__()
        self.in_channels = in_channels
        self.out_channels = out_channels
        if in_channels == 3 or in_channels == 9:
            self.rel_channels = 8
            self.mid_channels = 16
        else:
            self.rel_channels = in_channels // rel_reduction
            self.mid_channels = in_channels // mid_reduction
        #self.conv1 = nn.Conv2d(self.in_channels, self.rel_channels, kernel_size=1)
        #self.conv2 = nn.Conv2d(self.in_channels, self.rel_channels, kernel_size=1)
        self.conv1 = nn.Conv2d(self.in_channels, self.out_channels//4, kernel_size=1)
        self.conv2 = nn.Conv2d(self.in_channels, self.out_channels//4, kernel_size=1)
        self.conv3 = nn.Conv2d(self.in_channels, self.out_channels//4, kernel_size=1)
        self.conv4 = nn.Conv2d(self.in_channels, self.out_channels//4, kernel_size=1)
        self.conv5 = nn.Conv2d(self.in_channels, self.out_channels//4, kernel_size=1)
```

```
        self.conv6 = nn.Conv2d(self.in_channels, self.out_channels//4, kernel_size=1)
        self.conv7 = nn.Conv2d(self.in_channels, self.rel_channels, kernel_size=1)
        self.conv8 = nn.Conv2d(self.in_channels, self.rel_channels, kernel_size=1)
        self.conv9 = nn.Conv2d(self.rel_channels, self.out_channels//4, kernel_size=1)
        self.tanh = nn.Tanh()
        for m in self.modules():
            if isinstance(m, nn.Conv2d):
                conv_init(m)
            elif isinstance(m, nn.BatchNorm2d):
                bn_init(m, 1)

def forward(self, x, A=None,A5=None, alpha=1,alpha1=1):

    #    print('T1',T1.shape,type(T1))
      # x3=self.conv3(x)
        #print('A',A.shape)
      # print('A5',A5.shape)
        #A1=A[0]

        #A2=A[1]
        x1=torch.einsum('vu,nctu->nctv', A, self.conv1(x))
      # print('x1',x1.shape)
        x7=torch.einsum('vu,nctu->nctv', A5, self.conv6(x))
        x7=(x1+x7*alpha)/2
        #print('x2',x2.shape)
        x5=self.conv5(x)
        x8=self.conv7(x).mean(-1)
        x9=self.conv8(x).mean(-2)
        x8 = self.tanh(x8.unsqueeze(-1) - x9.unsqueeze(-2))
        x8=torch.einsum('vu,nctu->nctv', A5, self.conv9(x8))
        x8 = torch.einsum('nctv,nctu->nctv', x8, x5)
        #print('x8',x8.shape)
        #print('x9',x9.shape)
        #x1 = torch.einsum('nctv,nctv->nctv', x4, x3)
        #v1-1t
        vt=self.tanh(A.float().mean(-2).unsqueeze(-2)-A5.float().mean(-1).unsqueeze(-1))
        #vt1=vt1.long().to('cuda:0')
        #vt2=self.tanh(A5.float().mean(-2).unsqueeze(-2)-A.float().mean(-1).unsqueeze(-1))
        #vt= torch.zeros(25, 25).to('cuda:0')
        #vt[:, 0] = vt1
        #vt[0, :] = vt2
        #x3=torch.mul(x1, vt1)
        x3=torch.einsum('uv,nctv->nctu', vt, self.conv3(x))
        #x3=torch.einsum('ncuv,nctu->nctv', x1, x3)
```

```
        # print('x5',x3.shape)
         #x4=torch.einsum('nctu,ncvu->nctv', x7, x4)
         x3=(x3+x7*alpha1)/2
        # print('x6',x4.shape)

        # print('x9',x5.shape)
         x1=torch.cat((x1,x7,x8,x3),dim=1)
        # print('x7',x1.shape)
         #print('A4',A5.shape)
         #A5=A5.unsqueeze(0).unsqueeze(0)
         #print('A5',A5.shape)
         #A5 = F.interpolate(A5, size=(64, 64), mode='bilinear', align_corners=False)
        # print('A6',x5.shape)
         #x1=torch.einsum('ut,nctv->ncvu', vt2, self.conv4(x))
         #x1=x1*alpha + A5    # N,C,V,V
         #print('x1',x1.shape)
         #print('x5',x5.shape)

         return x1

class unit_tcn(nn.Module):
    def __init__(self, in_channels, out_channels, kernel_size=9, stride=1):
        super(unit_tcn, self).__init__()
        pad = int((kernel_size - 1) / 2)
        self.conv = nn.Conv2d(in_channels, out_channels, kernel_size=(kernel_size, 1),
padding=(pad, 0),
                              stride=(stride, 1))

        self.bn = nn.BatchNorm2d(out_channels)
        self.relu = nn.ReLU(inplace=True)
        conv_init(self.conv)
        bn_init(self.bn, 1)

    def forward(self, x):
        x = self.bn(self.conv(x))
        return x


#Spatial model
class unit_gcn(nn.Module):
    def __init__(self, in_channels, out_channels, A, A5,coff_embedding=4, adaptive=True,
residual=True):
        super(unit_gcn, self).__init__()
        inter_channels = out_channels // coff_embedding
```

```
        self.inter_c = inter_channels
        self.out_c = out_channels
        self.in_c = in_channels
        self.adaptive = adaptive
        self.num_subset = A.shape[0]
        self.convs = nn.ModuleList()
        for i in range(self.num_subset):
            self.convs.append(CTRGC(in_channels, out_channels))

        if residual:
            if in_channels != out_channels:
                self.down = nn.Sequential(
                    nn.Conv2d(in_channels, out_channels, 1),
                    nn.BatchNorm2d(out_channels)
                )
            else:
                self.down = lambda x: x
        else:
            self.down = lambda x: 0
        if self.adaptive:
            self.PA = nn.Parameter(torch.from_numpy(A.astype(np.float32)))
        else:
            self.A = Variable(torch.from_numpy(A.astype(np.float32)), requires_grad=False)
        self.A5=Variable(torch.from_numpy(A5.astype(np.float32)), requires_grad=False)
        self.alpha = nn.Parameter(torch.zeros(1))
        self.alpha1 = nn.Parameter(torch.zeros(1))
        self.bn = nn.BatchNorm2d(out_channels)
        self.soft = nn.Softmax(-2)
        self.relu = nn.ReLU(inplace=True)

        for m in self.modules():
            if isinstance(m, nn.Conv2d):
                conv_init(m)
            elif isinstance(m, nn.BatchNorm2d):
                bn_init(m, 1)
        bn_init(self.bn, 1e-6)

    def forward(self, x):
        y = None
        if self.adaptive:
            A = self.PA
        else:
            A = self.A.cuda(x.get_device())
        A5=self.A5.cuda(x.get_device())
```

```
        for i in range(self.num_subset):
            z = self.convs[i](x, A[i],A5, self.alpha,self.alpha1)
            y = z + y if y is not None else z
        y = self.bn(y)
        #y = self.relu(y)

#        xa=torch.stack((x,x),dim=1)
       # a,b,c,d,e=xa.size()
     #    x=xa.view(a*b,c,d,e)
       # print('x',x.shape)
       # print('y',y.shape)
        y = self.down(x)+y
        y = self.relu(y)

        return y

#Temporal model
class TCN_GCN_unit(nn.Module):
    def __init__(self, in_channels, out_channels, A, A5,stride=1, residual=True, adaptive=True,
kernel_size=5, dilations=[1,2]):
        super(TCN_GCN_unit, self).__init__()
        self.gcn1 = unit_gcn(in_channels, out_channels, A,A5, adaptive=adaptive)

        #self.se = SELayer(out_channels)   # Add SELayer here

        self.tcn1 = MultiScale_TemporalConv(out_channels, out_channels,
kernel_size=kernel_size, stride=stride, dilations=dilations,
                                            residual=False)
        self.relu = nn.ReLU(inplace=True)
        if not residual:
            self.residual = lambda x: 0

        elif (in_channels == out_channels) and (stride == 1):
            self.residual = lambda x: x

        else:
            self.residual = unit_tcn(in_channels, out_channels, kernel_size=1, stride=stride)

    def forward(self, x):
        #print("Input shape: ", x.shape)
       # y = self.gcn1(x)
        #print("After gcn1 shape: ", y.shape)
       # y = self.se(y)   # Apply SELayer here
        #print("After SELayer shape: ",y.shape)
```

```
        # y = self.relu(self.tcn1(y)) + self.residual(x)
        y = self.relu(self.tcn1(self.gcn1(x))) + self.residual(x)
        return y

#ST-HCN
class Model0(nn.Module):
    def __init__(self, num_class=120, num_point=18, num_person=2, graph=None, graph_args=dict(), in_channels=3,
                 drop_out=0, adaptive=True):
        super(Model0, self).__init__()

        if graph is None:
            raise ValueError()
        else:
            Graph = import_class(graph)
            self.graph = Graph(**graph_args)

        A = self.graph.A # 3,25,25
        #A=np.ones((3,25,25))
        G1 = hgut.generate_G_from_H(A[0])+hgut.generate_G_from_H(A[0].T)
        G2 = hgut.generate_G_from_H(A[1])+hgut.generate_G_from_H(A[1].T)
        #G3 = hgut.generate_G_from_H(A[2])+hgut.generate_G_from_H(A[2].T)
        A1=np.expand_dims(G1,axis=0)
        A2=np.expand_dims(G2,axis=0)
        #A3=np.expand_dims(G3,axis=0)
        A5=A[2]
        A = np.concatenate((A1, A2))
        #print('G',A.shape)
        self.num_class = num_class
        self.num_point = num_point
        #self.data_bn = nn.BatchNorm1d(int(num_person * in_channels * num_point * 2 / 5)) #* 2 / 5
        self.data_bn = nn.BatchNorm1d(num_person * in_channels * num_point)

        base_channel = 64
        self.l1 = TCN_GCN_unit(in_channels, base_channel, A,A5, residual=False, adaptive=adaptive)
        self.l2 = TCN_GCN_unit(base_channel, base_channel, A,A5, adaptive=adaptive)
        self.l3 = TCN_GCN_unit(base_channel, base_channel, A, A5,adaptive=adaptive)
        self.l4 = TCN_GCN_unit(base_channel, base_channel, A,A5, adaptive=adaptive)
        self.l5 = TCN_GCN_unit(base_channel, base_channel*2, A, A5,stride=2, adaptive=adaptive)
        self.l6 = TCN_GCN_unit(base_channel*2, base_channel*2, A,A5, adaptive=adaptive)
        self.l7 = TCN_GCN_unit(base_channel*2, base_channel*2, A, A5,adaptive=adaptive)
```

```
        self.l8 = TCN_GCN_unit(base_channel*2, base_channel*4, A, A5,stride=2, adaptive=adaptive)
        self.l9 = TCN_GCN_unit(base_channel*4, base_channel*4, A,A5, adaptive=adaptive)
        self.l10 = TCN_GCN_unit(base_channel*4, base_channel*4, A,A5, adaptive=adaptive)

        self.fc = nn.Linear(base_channel*4, num_class)
        nn.init.normal_(self.fc.weight, 0, math.sqrt(2. / num_class))
        bn_init(self.data_bn, 1)
        if drop_out:
            self.drop_out = nn.Dropout(drop_out)
        else:
            self.drop_out = lambda x: x

    def forward(self, x):
        if len(x.shape) == 3:
            N, T, VC = x.shape

            x = x.view(N, T, self.num_point, -1).permute(0, 3, 1, 2).contiguous().unsqueeze(-1)
        N, C, T, V, M = x.size()

        x = x.permute(0, 4, 3, 1, 2).contiguous().view(N, M * V * C, T)

        x = self.data_bn(x)
        x = x.view(N, M, V, C, T).permute(0, 1, 3, 4, 2).contiguous().view(N * M, C, T, V)
        x = self.l1(x)
        x = self.l2(x)
        x = self.l3(x)
        x = self.l4(x)
        x = self.l5(x)
        x = self.l6(x)
        x = self.l7(x)
        x = self.l8(x)
        x = self.l9(x)
        x = self.l10(x)

        # N*M,C,T,V
        c_new = x.size(1)
        x = x.view(N, M, c_new, -1)
        x = x.mean(3).mean(1)
        x = self.drop_out(x)

        return self.fc(x)

#CD-HCN
```

```
class Model1(nn.Module):
    def __init__(self, num_class=120, num_point=18, num_person=2, graph=None, graph_args=dict(), in_channels=3,
                 drop_out=0, adaptive=True):
        super(Model1, self).__init__()

        if graph is None:
            raise ValueError()
        else:
            Graph = import_class(graph)
            self.graph = Graph(**graph_args)

        A = self.graph.A # 3,25,25
        #A=np.ones((3,25,25))
        G1 = hgut.generate_G_from_H(A[0])+hgut.generate_G_from_H(A[0].T)
        G2 = hgut.generate_G_from_H(A[1])+hgut.generate_G_from_H(A[1].T)
        #G3 = hgut.generate_G_from_H(A[2])+hgut.generate_G_from_H(A[2].T)
        A1=np.expand_dims(G1,axis=0)
        A2=np.expand_dims(G2,axis=0)
        #A3=np.expand_dims(G3,axis=0)
        A5=A[2]
        A = np.concatenate((A1, A2))
        #print('G',A.shape)
        self.num_class = num_class
        self.num_point = num_point
        #self.data_bn = nn.BatchNorm1d(int(num_person * in_channels * num_point * 2 / 5))
        self.data_bn = nn.BatchNorm1d(num_person * in_channels * num_point)

        base_channel = 64
        self.l1 = TCN_GCN_unit(in_channels, base_channel, A,A5, residual=False, adaptive=adaptive)
        self.l2 = TCN_GCN_unit(base_channel, base_channel, A,A5, adaptive=adaptive)
        self.l3 = TCN_GCN_unit(base_channel, base_channel, A, A5,adaptive=adaptive)
        self.l4 = TCN_GCN_unit(base_channel, base_channel, A,A5, adaptive=adaptive)
        self.l5 = TCN_GCN_unit(base_channel, base_channel*2, A, A5,stride=2, adaptive=adaptive)
        self.l6 = TCN_GCN_unit(base_channel*2, base_channel*2, A,A5, adaptive=adaptive)
        self.l7 = TCN_GCN_unit(base_channel*2, base_channel*2, A, A5,adaptive=adaptive)
        self.l8 = TCN_GCN_unit(base_channel*2, base_channel*4, A, A5,stride=2, adaptive=adaptive)
        self.l9 = TCN_GCN_unit(base_channel*4, base_channel*4, A,A5, adaptive=adaptive)
        self.l10 = TCN_GCN_unit(base_channel*4, base_channel*4, A,A5, adaptive=adaptive)

        self.fc = nn.Linear(base_channel*4, num_class)
```

```
        nn.init.normal_(self.fc.weight, 0, math.sqrt(2. / num_class))
        bn_init(self.data_bn, 1)
        if drop_out:
            self.drop_out = nn.Dropout(drop_out)
        else:
            self.drop_out = lambda x: x

    def forward(self, x):
        if len(x.shape) == 3:
            N, T, VC = x.shape
            x = x.view(N, T, self.num_point, -1).permute(0, 3, 1, 2).contiguous().unsqueeze(-1)
        N, C, T, V, M = x.size()

        x = x.permute(0, 4, 3, 1, 2).contiguous().view(N, M * V * C, T)
        x = self.data_bn(x)
        x = x.view(N, M, V, C, T).permute(0, 1, 3, 4, 2).contiguous().view(N * M, C, T, V)
        x = self.l1(x)
        x = self.l2(x)
        x = self.l3(x)
        x = self.l4(x)
        x = self.l5(x)
        x = self.l6(x)
        x = self.l7(x)
        x = self.l8(x)
        x = self.l9(x)
        x = self.l10(x)

        # N*M,C,T,V
        c_new = x.size(1)
        x = x.view(N, M, c_new, -1)
        x = x.mean(3).mean(1)
        x = self.drop_out(x)

        return self.fc(x)


# Channel differential mechanism
class Model(nn.Module):
    def __init__(self, num_class=120, num_point=18, num_person=2, graph=None, graph_args=dict(), in_channels=3,
                 drop_out=0, adaptive=True):
        super(Model, self).__init__()

        self.model0=Model0(graph=graph)
```

```
        self.model1=Model1(graph=graph)
    def forward(self, x):
        x1=x
        x[:,0,:,:,:]=((x1[:,0,:,:,:]-x1[:,1,:,:,:])+(x1[:,0,:,:,:]-x1[:,2,:,:,:]))/2
        x[:,1,:,:,:]=((x1[:,1,:,:,:]-x1[:,2,:,:,:])+(x1[:,1,:,:,:]-x1[:,0,:,:,:]))/2
        x[:,2,:,:,:]=((x1[:,2,:,:,:]-x1[:,0,:,:,:])+(x1[:,2,:,:,:]-x1[:,1,:,:,:]))/2
        out0=self.model0(x1)
        out1=self.model1(x)

        #return out1
        return out0,out1
```
