# Supplementary material for: Dual-branch differential channel hypergraph convolutional network for human skeleton based action recognition
Source: PLoS One. 2025 Oct 27;20(10):e0332066. doi: 10.1371/journal.pone.0332066 (PMC12558502; doi:10.1371/journal.pone.0332066)
Supplement: Attachment — Submitted filename: PLONE review PONE-D-25-23177.docx [file pone.0332066.s002.docx]

This paper proposed a dual-branch differential channel hypergraph convolutional neural network to address the challenge of incomplete representation of non-natural dependencies in skeletal structure for skeleton-based human action recognition. The proposed model is evaluated on three public datasets to demonstrate its effectiveness.

However, several areas in this paper need further improvement to enhance its quality:

1. The most typical improvement in metric is suggested to add to the abstract in order to validate the superiority of the proposed method in this paper.
2. The references should include more relevant and recent works. The introduction should contain more comprehensive review of recent studies.
3. Some cited reference numbers in the text are incorrectly shown. For example, on Page 2 of this paper, “Yan et al. Error! Reference source not found”, Page 3, “Chen et al. Error! Reference source not found”. And some references do not match the cited numbers in the text. For example, Page 5, “developed by Yan et al.[1]” . Actually, Ref. [1] is not written by Yan et al.
4. The title of each section should be numbered.
5. The format of the text should meticulously checked. For example, “where” followed by a equation does not need indentation, etc. In the text, some fonts about figures and tables like “ Fig.3(b)” should be corrected.
6. In the comparative experiment, it should add more comparison with the state-of-the-art methods. And the results of the existing models in Table 5 should be clarified whether they are cited from the references or are run in the settings of this paper. Furthermore, each existing model is better to be labeled the corresponding number of reference.
7. It is recommended to use more diagrams to demonstrate the effectiveness of the proposed method. For instance, the accuracy curve varying with some hyper-parameters, the accuracy histogram of each category, and some visualization plots for depicting the attention weights of each skeleton joints in each frame.
8. Some figures like the confusion matrices in Fig. 6 are blurry, which do not clearly show the ratio of correct or wrong classification, and the color variance for each square of predicted label-true label. In addition, the accuracy values like 90%, 59.17%, 80%, 8183% in the following analysis can not be found in all the figures and tables.
9. In conclusion, the limitation of this paper should be discussed and the future work should be planned.
10. The model name is sometimes called “BDC-HCN”, sometime called “DBC-HCN”. Please correct this discrepancy.
11. Some full names of the key terms are printed in lowercase, some others are capitalized.
12. In Fig. 2(b), it seems that there are some mistakes in the feature matrix. For example, it should not be 1 but 0 for n1-e3.
13. All the acronyms should be accompanied with the full names in the first appearance of the corresponding terms, then the acronyms can be individually used.
